# Surgical Treatment of Intra‐Articular Distal Humeral Fractures by using a Combined Medial and Lateral Approach: An Anatomic Study

**DOI:** 10.1111/os.12475

**Published:** 2019-06-17

**Authors:** Li‐biao Wei, Tu Hu, Jie Liu, Zhi‐quan An

**Affiliations:** ^1^ Department III of Traumatic Orthopedics Surgery Shanghai Jiao Tong University Affiliated Sixth People's Hospital Shanghai China; ^2^ Library of Shanghai Jiao Tong University School of Medicine Shanghai China

**Keywords:** Articular surface, Combined medial‐lateral approaches, Elbow joint, Exposure, Olecranon osteotomy, Surgical approach

## Abstract

**Objective:**

To determine the visible size of the distal humeral articular surface by using a novel combined medial‐lateral approach as an alternative method of surgical treatment for intra‐articular distal humeral fractures.

**Methods:**

In this anatomical study, 12 adult fresh‐frozen cadaveric elbows were randomly divided into a medial‐lateral group and an olecranon osteotomy group, with 6 in each group. In the medial‐lateral group, a medial approach was first used, and then a lateral approach. The sizes of the distal humeral articular surface exposed by each incision and the joined size were measured and calculated. In the olecranon osteotomy group, a posterior olecranon osteotomy approach was applied, and the maximal visible sizes of the articular surface were marked and calculated. Ratios of the maximal sizes of the distal humeral articular surface of the two approaches were compared.

**Results:**

In the medial‐lateral group, the medial approach could expose 2/5 of the medial trochlea and 1/3 of the capitellum, while the mean visible size of the distal humeral articular surface was 6.8 cm^2^, 34.8% of the entire surface; the lateral approach can expose 3/7 of the capitellum and 1/4 of the medial trochlea, while the mean visible size of the distal humeral articular surface was 6.7 cm^2^, 33.9% of the whole surface; for the combined medial‐lateral approach, the mean scope exposed of the medial and lateral visible articular surface was 38.2% and 43.1%, respectively. Meanwhile, in the olecranon osteotomy group, the posterior olecranon osteotomy was found to expose most of the posterior distal humeral articular surface, except for 1/3 of the anterior trochlea and 1/4 of the anterior capitellum, and the visible range of articular surface was 65.3%. The combined medial‐lateral approach exposed 9.2 cm^2^ in total, 46.9% of the whole distal humeral articular surface, which averaged 19.6 cm^2^. However, the visible size of the distal humeral articular surface for the olecranon osteotomy approach was 13.7 cm^2^, 63.1% of the entire distal humeral articular surface, which averaged 21.3 cm^2^. There was a significant difference observed between the medial‐lateral group (46.9%) and the olecranon osteotomy group (63.1%) for the maximal visible size of the distal humeral articular surface (*t* = 7.201, *P* = 0.001).

**Conclusions:**

The combined medial‐lateral approach can expose 46.9% of the distal humeral articular surface, concentrating on the anterior part, so it can be recommended to treat intra‐articular fractures with a simple pattern in the posterior with the anterior side of the distal humerus less comminuted.

## Introduction

Distal humeral fractures comprise 1%–7% of all fractures and 30% of all elbow fractures. Intercondylar fractures of the distal humerus make up approximately 2% of all fractures^1^. Distal humeral fractures are clinically difficult to deal with, especially intra‐articular fractures, which are among the most complicated fractures^2^, ^3^. Anatomically, the distal humerus has a triangular shape, which consists of two columns and a “tie arch”. The medial column holds at its distal end the nonarticular medial epicondyle with the insertion of flexor muscles and the medial humeral trochlea. The lateral column holds at its distal end the capitellum and more proximally the lateral epicondyle with the insertion of extensor muscles. From a lateral perspective, the articular surface of the trochlea and capitellum is projected anteriorly at an angle of 40° to the axis of the humerus, the trochlea axis being externally rotated at an angle of 3° to 8° and compared with the longitudinal axis being in 4° to 8° of valgus. Anatomic reconstruction of the articular surface and stable internal fixation are key factors of the excellent functional effects^4^. For the purpose of good anatomic construction and stable internal fixation, it is essential to gain enough exposure of the articular surface.

Displaced intra‐articular distal humeral fractures usually need to be treated with open reduction and internal fixation (ORIF)^5^, ^6^. Many approaches are reported in the literature on surgical treatment of this fracture pattern^7^, among which the posterior olecranon osteotomy approach is considered to be a better one, with the advantage of providing more optimal visualization of distal articular surfaces^8^, ^9^, ^10^, and despite significant complications such as delayed union, non‐union, heterotrophic ossification, ulnar nerve paralysis, symptomatic olecranon fixation, and secondary procedures being required for the removal of symptomatic hardware having been reported^9^, ^11^. It is reported that intra‐articular distal humeral fractures could be treated with ORIF through a combined medial‐lateral elbow approach^12^. The medial articular fragment of the distal humerus is first reduced to the medial column by a medial elbow approach, and the lateral fragment is then reduced to the medial fragment and lateral column through a lateral elbow approach. A contoured reconstructive plate is placed on the anteromedial and anterolateral side of the distal humerus, respectively. This approach has the advantage of keeping the integrity of the elbow extensor and, therefore, does not affect the extension power of the elbow. It is also found that this approach is useful in surgical treatment of AO/OTA C1 and C2, with a simple fracture pattern in the posterior distal humerus. However, until now, there has been no anatomical research regarding the maximal visible size of the distal humeral articular surface that could be identified when this combined medial‐lateral elbow approach was applied to treat intra‐articular fractures of the distal humerus.

First, this anatomical study is designed to determine the exposed scope of the articular surface and the maximal visible sizes of the distal humeral articular surface to be exposed in the combined medial‐lateral approach in open reduction of intra‐articular distal humeral fractures. Second, it can be concluded the indications of the combined medial‐lateral approach so that we can use the approach more reasonably. Third, comparing the combined medial‐lateral approach with the olecranon osteotomy approach, distinctions of the articular scope and the maximal visible sizes of the distal humeral articular surface to be exposed can be determined so that the two approaches in the treatment of intra‐articular distal humeral fractures will provide a better choice.

## Materials and Methods

### *Inclusion and Exclusion Criteria*

Twelve adult fresh‐frozen cadaveric upper extremities were enrolled in this study. All the extremities that met the following criteria were included: (i) each extremity consisted of segments from the shoulder to the hand; and (ii) each extremity was intact apart from being stripped of skin and underlying fascia. All the extremities were excluded based on the following criteria: (i) accompanying deformity; (ii) with significant joint degeneration; and (iii) any previous dissection of deep structures. The study was approved by the Medical Ethical Committee of the Shanghai Sixth People's Hospital and has, therefore, been performed in accordance with the ethical standards laid down in the 1964 Declaration of Helsinki and its later amendments. The 12 elbows were randomly divided into two groups: the medial‐lateral group and the olecranon osteotomy group, 6 in each.

### *Surgical Technique*

**Figure 1:**
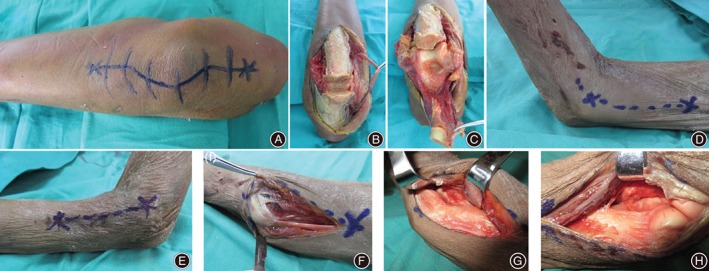
Major procedures of the two surgical approaches to the elbow: Olecranon osteotomy (A–C) and combined medial‐lateral approaches (D–H).

**Figure 2:**
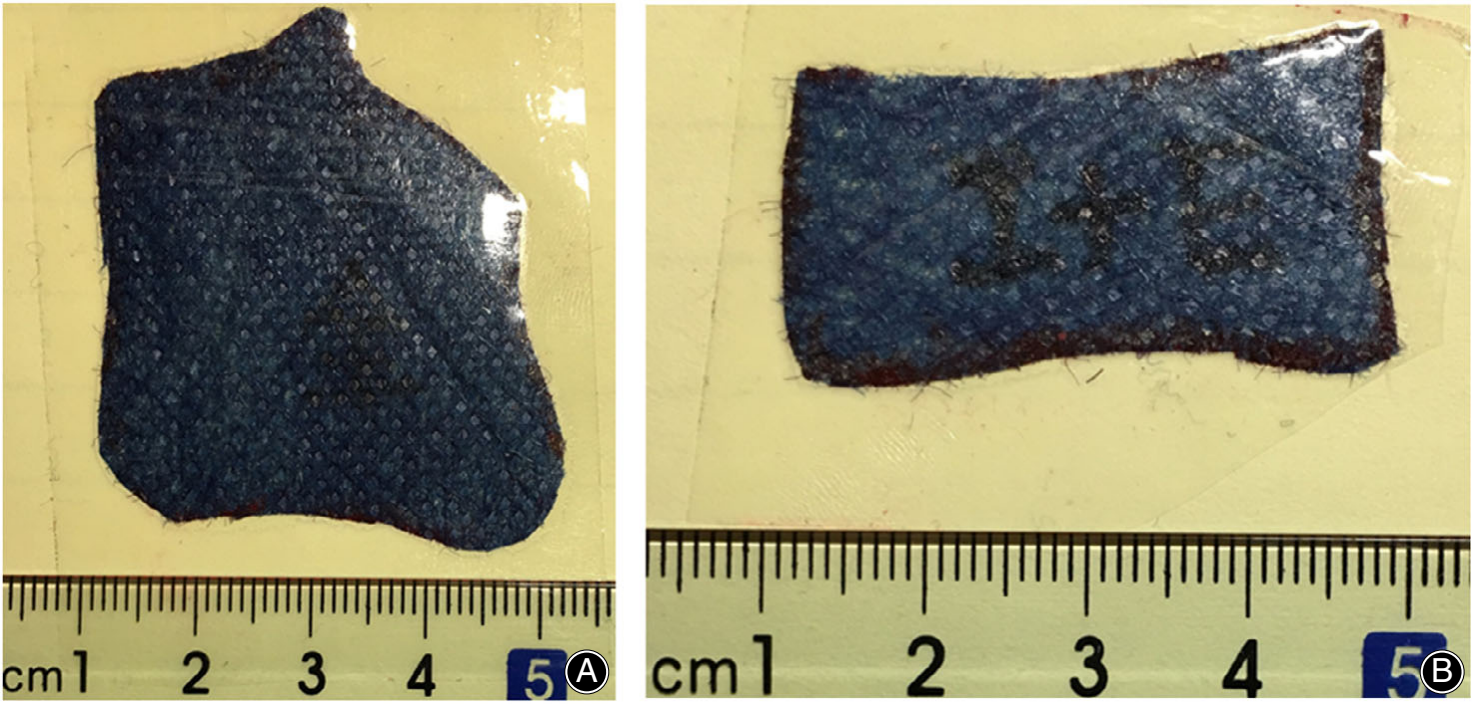
The photograph with a standard scale: A, one of the entire distal humeral articular surface facsimiled with a blue soft cloth; B, one of the visible sizes of distal humeral articular surface facsimiled with a blue soft cloth.

In the medial‐lateral group, dissection was performed according to the literature^11^. The fresh‐frozen cadaveric upper extremities stayed in the position of palm up. The medial approach from medial epicondyle to proximal humerus approximately 7 cm with the elbow flexed approximately 60° was first used. The common flexor tendon was partly divided and the capsule was dissected to expose the articular surface. The elbow was passively flexed and extended while the soft tissue was retracted anteriorly. The borderline of the maximal visible articular surface was identified and marked first with a 1.5‐mm Kirschner wire which was used as a drill bit and then painted with a red marker pen. The medial incision was then sutured. Next, the lateral approach was initiated from the lateral epicondyle to the proximal humerus, approximately 8 cm. The brachial muscle was stripped from the anterolateral side of the distal humerus and the common extensor tendon was partly dissected to expose the articular surface. The articular surface borderline was identified at the elbow flexion and extension in a similar fashion as the medial side except that the color of the marker pen is blue. The elbow was dislocated and all the soft tissue attachments were dissected away to expose the entire distal humerus (Fig. 1). The marked area of the medial, the lateral, the joined area of the visible marked articular surface, and the entire distal humeral articular surface were facsimiled with a blue soft cloth, respectively. The clothes were cut and then photographed using a standard scale, and the sizes and range were calculated by using the software Adobe Photoshop CS6 (Adobe, USA) according to the comparison of pixel value. The percentage of the visible distal humeral articular surface of the entire distal humeral surface was calculated, and the range of medial and lateral was transformed into the percentage of the exposed length to the whole length of humeral trochlea and capitellum, which was a curve scope virtually (Fig. 2).

In the olecranon osteotomy group, the fresh‐frozen cadaveric upper extremities stayed in the position of palm down with the arm supported over a bolster. A posterior mid‐incision was made first. Next, medial and lateral skin flaps were elevated, with care taken to protect cutaneous nerve branches and keep them within the skin flaps. The ulnar nerve was recognized by the medial border of the triceps, dissected at least 6 cm proximally and distally, and left in an anteriorly transposed position in the subcutaneous tissues. The osteotomy was proceeded approximately 2 cm distal to the tip of the olecranon. After the olecranon osteotomy was finished, the posterior elbow capsule was dissected and the distal humeral articular surface was exposed (Fig. 1). The visible area, the range, and the entire distal humeral articular surface were marked, facsimiled, measured, and calculated in the same manner as for the medial‐lateral group.

### *Statistical Analysis*

The quantitative data included in the study were sizes, ranges, and percentages. Sizes of the medial, lateral, medial‐lateral, and the whole distal articular surface were measured and percentages of the first three to the whole were calculated. The range of exposed articular surface was transformed into the percentage of the exposed length to the whole length of the distal humeral articular surface. In the medial‐lateral group, the range and size of the visible area and its percentage of the whole were determined. In the olecranon osteotomy group, the above parameters were also calculated.

All these data were processed with the statistical software SPSS V20.0 (IBM company, USA). In addition, ratios of the maximal visible sizes of the distal humeral articular surface of the two approaches were compared using Student's *t*‐test.

Based on the calculated sizes and range, we can determine the exposed articular surface scope and the maximal visible sizes of the distal humeral articular surface, and we can confirm the indications of the combined medial‐lateral approach, which can provide a better choice of surgical approach in the treatment of intra‐articular distal humeral fractures.

## Results

### *General Results for the Exposed Articular Surface Range*

When the medial approach was used, 2/5 of the anterior medial trochlea and 1/3 of the anterior capitellum scope were visible, and when the lateral approach was used independently, almost 3/7 of the anterior capitellum and 1/4 of the anterior trochlea scope could be identified under direct division. While the combined medial‐lateral approach was used, the mean range of the medial visible articular surface was 38.2% (range, 30%–46.3%) of the entire surface of the distal humerus, and the mean visible range of the lateral articular surface that could be identified was 43.1% (range, 32.7%–49.2%) of the whole distal articular surface (Table 1). When the posterior olecranon osteotomy approach was applied, most of the posterior articular surface was exposed, except 1/3 of the anterior medial trochlea and 1/4 of the anterior capitellum, and the visible range of the articular surface was 65.3% (range, 55.8%–76.4%) of the whole distal articular surface (Table 2). It could be concluded that the visible scope of the combined medial‐lateral approach mainly focused on the anterior trochlea and capitellum, but it mainly concentrated on the posterior distal humeral articular surface for the olecranon osteotomy approach.

**Table 1:** Data of the combined medial‐lateral approach (medial‐lateral group)

Parameters	No. 1	No. 2	No. 3	No. 4	No. 5	No. 6	Mean
Medial (cm^2^)	6.14	4.9	5.05	7.99	9.177	7.625	6.8
Lateral (cm^2^)	6.892	4.144	4.658	7.96	9.164	7.513	6.7
Medial‐lateral (cm^2^)	10.988	6.701	7.362	8.846	12.613	8.789	9.2
Whole distal articular surface (cm^2^)	22.358	14.339	15.703	20.246	26.388	18.668	19.6
Medial/total (%)	27.5	34.2	32.2	39.5	34.8	40.8	34.8
Lateral/total (%)	30.8	28.9	29.7	39.3	34.7	40.2	33.9
Medial‐lateral/total (%)	49.1	46.7	46.9	43.7	47.8	47.1	46.9
Range of medial (%)	30	34.7	32.1	45.6	46.3	40.5	38.2
Range of lateral (%)	46.9	43.6	32.7	49.2	40.9	45.1	43.1

**Table 2:** Data of the posterior olecranon osteotomy approach (olecranon osteotomy group)

Parameters	No. 1	No. 2	No. 3	No. 4	No. 5	No. 6	Mean
Visible size (cm^2^)	15.037	14.656	16.446	9.69	11.518	13.473	13.5
Whole distal articular surface (cm^2^)	20.665	23.826	25.668	16.797	19.072	21.76	21.3
Visible size/total (%)	72.8	61.5	64.1	57.7	60.4	61.9	63.1
Visible range (%)	76.4	55.8	66.7	59.6	64.4	69	65.3

### *General Results for the Visible Sizes of the Articular Surface*

**Figure 3:**
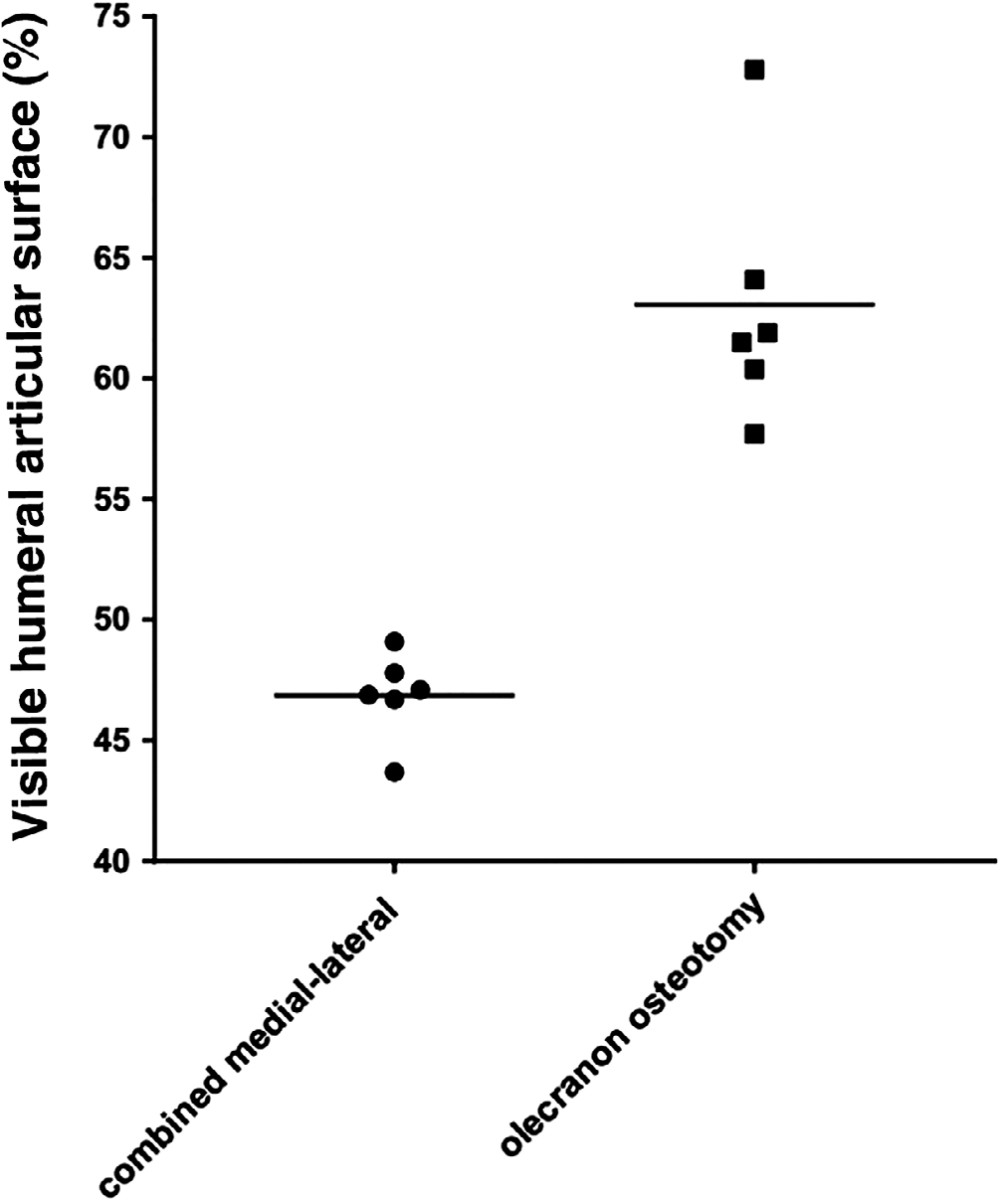
Scattergraph showing percentage of visible distal humeral articular surface: combined medial‐lateral approaches (circles) and olecranon osteotomy (squares), the *linear transverse or horizontal line* equal to mean.

**Figure 4:**
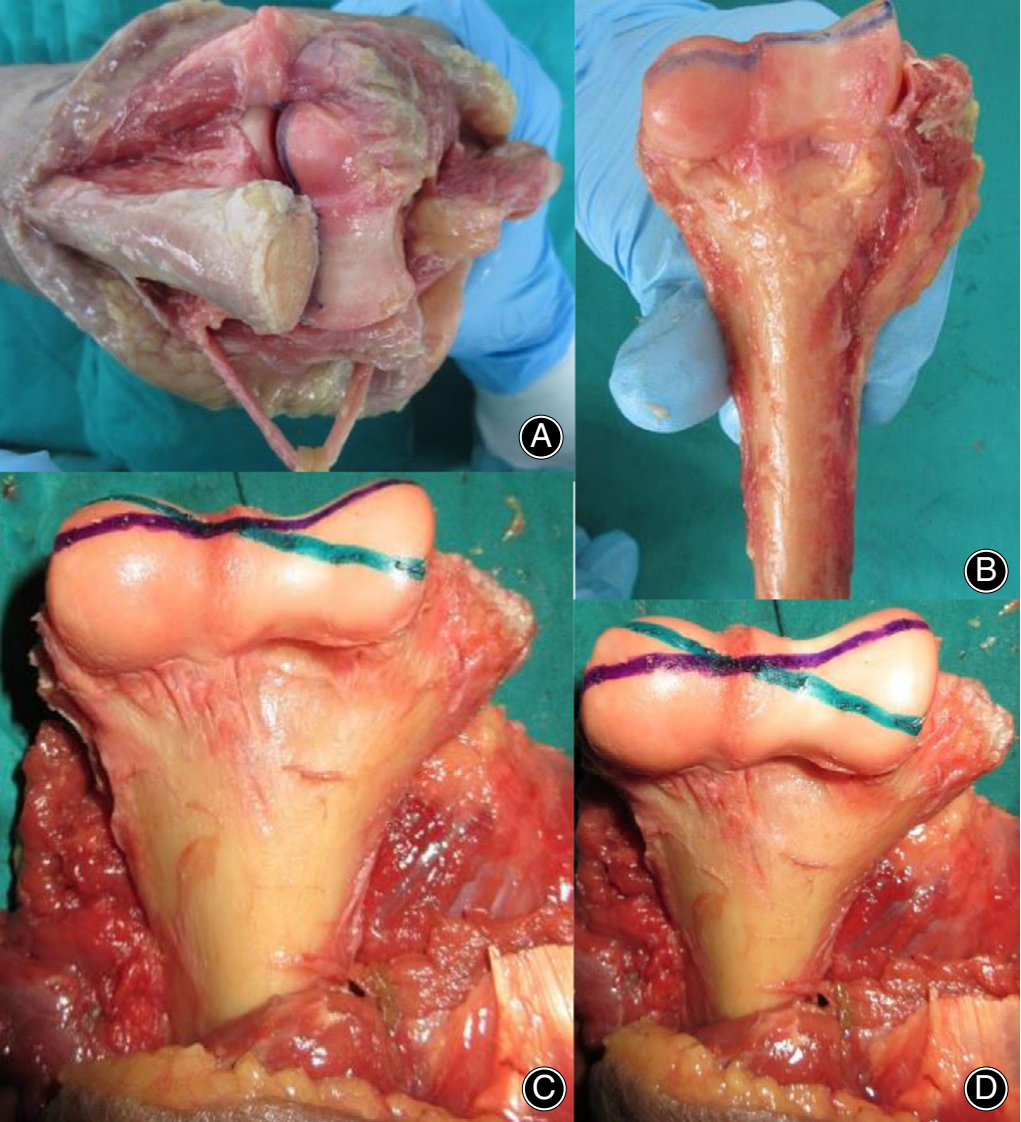
Photograph marked by marking pen showing the exposure of distal humeral articular surface of the two surgical approaches: olecranon osteotomy (A,B) and combined medial‐lateral approaches (C,D), with the painted margin equal to the visible distal humeral articular surface.

For the medial approach, the mean size of the visible articular surface was 6.8 cm^2^ (range, 4.9–9.2 cm^2^), 34.8% (range, 27.5%–40.8%) of the entire surface of the distal humerus, and for the lateral approach, the mean visible size of the articular surface that could be identified was 6.7 cm^2^ (range, 4.1–9.2 cm^2^), 33.9% (range, 28.9%–40.2%) of the whole distal articular surface. The maximal mean visible size of the distal humeral articular surface was 9.2 cm^2^ (range, 6.7–12.6 cm^2^), 46.9% (range, 43.7%–49.1%) of the entire distal humeral articular surface, 19.6 cm^2^ (range, 14.3–26.4 cm^2^) in the combined medial‐lateral approach, and was 13.7 cm^2^ (range, 9.7–16.4 cm^2^), 63.1% (range, 57.7%–72.8%) of the entire articular surface of distal humerus, 21.2 cm^2^ (range, 16.8–25.7 cm^2^) in the olecranon osteotomy approach (Tables 1 and 2, Fig. 3). There was a significant difference observed between the medial‐lateral group and the olecranon osteotomy group for the maximal visible size percentage of the distal humeral articular surface (46.9% and 63.1%, respectively; *t* = 7.201, *P* = 0.001). It could be inferred that the olecranon osteotomy exposed a larger articular surface, located mainly on the posterior side, than the combined medial‐lateral approach, which could provide good access to the anterior articular surface of the trochlea and the capitellum (Fig. 4).

## Discussion

Various approaches have been reported in the surgical treatment of intra‐articular distal humerus fractures, including triceps splitting^13^, triceps reflecting^14^, and olecranon osteotomy approaches^15^. Each has its advantages and disadvantages, but which is the best has not been confirmed. Wilkinson *et al*.^10^ reported that the exposed distal humeral articular surface percentage of the triceps splitting, the triceps reflecting, and the olecranon osteotomy approaches was 35%, 46%, and 57% in their study and the olecranon osteotomy provided good access to the posterior and inferior articular surfaces of the trochlea and the capitellum. All these approaches could not expose most of the anterior part of the distal articular surface and meanwhile affect the strength of the extensor. Our clinical research has demonstrated that some of the intra‐articular distal humerus fractures could be reduced and fixed through the combined medial‐lateral approach. However, there has been no anatomical study regarding the identifiable size of the articular surface when this approach has been applied. In this study, six adult fresh‐frozen cadaveric upper extremities were enrolled and the sizes of articular surfaces were measured when the combined medial‐lateral approach was used on the elbow. When the combined medial‐lateral approach was applied, the mean size of the joint surface was 9.2 cm^2^ (range, 6.7–12.6 cm^2^), 46.9% (range, 43%–49%) of the whole size of the entire distal humeral articular surface, which was 19.6 cm^2^ (range, 14.3–26.4 cm^2^). The combined medial‐lateral approach could provide good access to the anterior articular surface of the trochlea and the capitellum.

The size of the articular surface that could be exposed through the posterior olecranon osteotomy approach on the six paired cadaveric upper extremities was also measured; 63.1% (range, 57.7%–72.8%) of the articular surface could be identified. This study demonstrated that although the combined medial‐lateral approach could not expose more articular surface than the posterior olecranon osteotomy approach, it could provide a better view of half of the distal humeral articular surface including the whole anterior articular surface. The exposure percentage of the distal humeral articular surface with the combined medial‐lateral approach (46.9%) in our study was more than that of the triceps splitting (35%) and triceps reflecting (46%) approach. This result was not reported previously. According to our results, this combined medial‐lateral approach is recommended to treat intra‐articular fractures with a simple pattern in the posterior with the anterior side of the distal humerus less comminuted. When the intra‐articular distal humerus fracture is severely comminuted in the articular surface, the olecranon osteotomy approach is recommended.

There are also several limitations in this study. First, the number of fresh‐frozen cadaveric elbows was not very large (12 totally), which could make the results unrepresentative. Second, the processes of mark and measure were not extremely precise, although the results calculated by Adobe Photoshop CS6 were accurate. In the future, the limitations will be reduced.

### *Conclusions*

This anatomical study demonstrated that compared with the posterior olecranon osteotomy approach, the combined medial‐lateral approach could expose 46.9% of the entire distal humeral articular surface located mainly on the anterior side of distal humerus, and it can be recommended to treat intra‐articular fractures with a simple pattern in the posterior with the anterior side of distal humerus less comminuted.
